# Pancreatic Cancer and Gastroenterology: A Review

**DOI:** 10.4021/gr563w

**Published:** 2013-07-14

**Authors:** Chijioke Enweluzo, Fahad Aziz

**Affiliations:** a Section on Hospital Medicine, Department of Internal Medicine, Wake Forest School of Medicine, Medical Center Boulevard, Winston Salem, NC 27101, USA

**Keywords:** Pancreatic cancer, Gastroenterology, Autoimmune pancreatitis, Chronic Pancreatitis

## Abstract

Pancreatic cancer is a well known aggressive and highly malignant condition with varied ways of presentation. It is the fourth commonest cause of cancer related deaths in the United States. Presenting symptoms and signs are closely related to tumor size and location. Imaging remains the most useful diagnostic modality and is typically applied in an “upgrade fashion” unless in the case of incidentally discovered pancreatic tumors. The role of gastroenterology in the diagnosis and treatment of patients with this condition has expanded recently and is expected to grow even more.

## Introduction

Pancreatic cancer is a condition that is usually diagnosed at an advanced stage with a myriad of presenting clues which are non specific and could be missed altogether if a high index of clinical suspicion is absent. Diagnosis especially of early stage pancreatic cancer is even more of a challenge as these tumors may be missed on imaging. This is where the role of gastroenterology is very important. This role does not stop here as therapeutic modalities like endoscopic resonance cholangiopancreaticography (ERCP) are increasing serving a therapeutic function in the care of these patients. This article seeks to take an overview of the presentation and diagnosis of this condition and the contribution made by gastroenterology in the care of these patients.

## Presentation

Pancreatic cancer is a well known aggressive and highly malignant condition with varied ways of presentation [1]. According to Hariharan et al it is the fourth most common cause of cancer related deaths in the United States and the eighth cause of the same worldwide [2]. Porta M et al, conducted a multi-center case series of 185 patients diagnosed with pancreatic cancer over a three-year period and found that 62 percent of these cases involved the head of the pancreas, 10 percent involved the body, 6 percent came from the tail, and the remainder were not specified [1]. In terms of primary presenting symptoms, they found that 79% of these patients complained of abdominal pain and 71% specifically described epigastric pain. Other symptoms included: asthenia, weight loss, anorexia, dark urine, jaundice, nausea, back pain, diarrhea, vomiting, steatorrhea and thrombophlebitis. Courvoisier’s sign, a well known and commonly described phenomenon where a non tender and gall bladder is palpable at the right coastal margin, was seen in 13% of patients in the same study [1]. Other studies have indicated that presenting symptoms and signs are closely related to the tumor size and location. These studies postulate that pancreatic head tumors commonly present with jaundice, steatorrhea, and weight loss, which are symptoms that are not commonly seen in tumors arising from the tail of the gland [3-5]. These symptoms are mainly secondary to biliary and pancreatic duct obstruction from the expanding tumor.

Signs of advanced disease include a large, easily palpable abdominal mass, significant weight loss, left supraclavicular node also known as Virchow’s node and palpable periumbilical mass also referred to as Sister Mary Joseph’s node. Trousseau’s sign in which there is thrombosis in the superficial and deep venous system anywhere in the body is also considered a very important physical sign suggestive of pancreatic cancer. Pannala R et al reported new onset diabetes mellitus as a presenting sign for pancreatic cancer [6]. The plethora of presenting signs and symptoms described above and their non-specificity make diagnosing this condition a herculean task; therefore, a high level of clinical suspicion is always helpful.

Pancreatic tumors are broadly divided into 2 groups: exocrine and endocrine. Adenocarcinomas make up majority of exocrine cancers with a high level of malignancy and poor prognosis. Mucinous pancreatic cancers are the second most common type of exocrine pancreatic cancers. They carry a slightly better prognosis compared to adenocarcinomas. Neuroendocrine tumors of the pancreas arise from the islet cells and account for a minimal portion of pancreatic tumors. They could be malignant. Another group known as cystic neoplasm of the pancreas is frequently encountered and possesses varying malignant potential across the group. An incidentally discovered pancreatic cyst may also be the first indication or concern for pancreatic malignancy. Goodman M et al conducted a study of 24 patients with incidentally discovered solid pancreatic lesions over 10 years and concluded that although uncommon, incidentally discovered solid pancreatic masses are malignant neoplasms which could be ductal adenocarcinomas or neuroendocrine tumors [7]. Risks factors can be hereditary or non hereditary. Hereditary risk factors include a family history of genetic syndromes that can increase cancer risk, including a BRCA2 gene mutation, Lynch syndrome and familial atypical mole-malignant melanoma (FAMMM) [8, 9]. A personal or family history of pancreatic cancer is also considered hereditary [9]. Non hereditary risk factors that have been proffered include black race, overweight/obesity, helicobacter pylori infection, chronic pancreatitis, diabetes mellitus and smoking. Diets low in fruits and vegetables, high in red meats, high in sugar or sweetened with fructose as used commonly in soda drinks have also been mentioned as possible risk factors [10, 11].

## Diagnosis

Imaging is the most useful initial diagnostic test. Abdominal ultrasound may be able to reveal tumors especially if there is concomitant biliary dilatation. The bigger the tumor, the better the sensitivity and specificity in this imaging modality. As in all forms of sonography, the expertise of the sonographer and radiologist reading the images contribute greatly to the validity of results. Abdominal CT preferably with contrast affords better sensitivity and specificity and is generally considered a step up from sonography. Again, imaging here is better enhanced with larger tumors [12]. Magnetic resonance cholangiopancreaticography (MRCP) is another useful imaging modality that is as sensitive as endoscopic resonance cholangiopancreaticography (ERCP) in evaluating the biliary and pancreatic ductal system without the need for contrast [13, 14]. Furthermore, it is actually the preferred modality in patients with bowel obstruction such as gastric outlet obstruction, duodenal stenosis or following surgical rearrangement like in Billroth II or Whipple procedure. Patients with biliary or pancreatic ductal obstruction as well as patients with unsuccessful ERCP should also be preferably evaluated by MRCP [15].

Speaking of ERCP, this is considered the diagnostic modality of choice. It allows direct visualization of the pancreaticobiliary system especially if done with pancreatoscopy. Niederau C et al conducted a metanalysis concluding with a sensitivity of 92% and specificity of 96% for diagnosing cancer of the pancreas by ERCP [16]. ERCP also provides the opportunity to collect tissue samples for histology. In the past the “double duct” sign on ERCP, which is due to an obstruction of the common bile and pancreatic ducts, was considered a pathognomic sign for pancreatic malignancy. However, Menges M et al in a case series of 43 patients identified as having a double duct stenosis on ERCP, 15% did not have pancreatic carcinoma [17]. This led to a conclusion that the specificity of the double duct sign in predicting the presence of pancreatic cancer was lower than previously reported. As lauded as it is, ERCP does not come without its limitations. High cost, the need for more skilled personnel, and relative unavailability across hospitals are widely known drawbacks. Potential complications such as bleeding, pancreatitis, gut perforation, cholangitis, and reactions to contrast dye are not uncommon and need to be weighed against the benefits of the procedure. Presently, the role of ERCP in pancreatic cancer is less diagnostic and more therapeutic especially in relieving biliary system obstruction by stenting. Endoscopic ultrasound fine needle aspiration (EUS-FNA) biopsy is more accurate in evaluating smaller tumors that may be missed on previously described modalities. It is also very useful in evaluating local metastasis especially to the liver and vascular invasion and hence staging of early disease. EUS-FNA biopsy is also considered the best option to obtain tissue for histology. This is because the risk of intra-peritoneal tumor spread is significantly reduced as the approach is through the bowel wall and not percutaneously. Other imaging modalities such as MRI, Chest CT, and PET scan are very useful in evaluating for distant metastasis and hence staging as this is very useful in guiding treatment. Staging laparoscopy has been found useful especially in surgically resectable disease [18, 19]. Laparoscopy also affords the opportunity to obtain peritoneal washings which if positive for malignancy are indicative of advanced disease. Preferred staging method for pancreatic cancer is the tumor-node-metastasis (TNM) system [19].

Besides imagining and obtaining tissue for histology, the serum marker CA-19 also helps in the diagnosis of pancreatic cancer especially in patients with a positive expression of the Lewis blood group antigen [20, 21]. In these individuals, sensitivity is better with bigger tumors as higher levels of the marker are often encountered [20, 21]. Even at that, present guidelines do not recommend using CA-19 as a screening test for pancreatic cancer due to its low and wide sensitivity, specificity and positive predictive value across the population as this marker can also be elevated in individuals with other hepatobiliary conditions [22]. It can definitely be used as a prognostic tool especially in monitoring response to treatment like in the post operative period following surgery for resectable pancreatic cancer [22].

In terms of differential diagnosis, chronic pancreatitis and autoimmune pancreatitis (AIP) are 2 conditions that are commonly mistaken for pancreatic cancer. Of course, pointers from history such as young age and prolonged alcohol abuse will favor a diagnosis of chronic pancreatitis while AIP can be suggested by positive serology results of IgG4 and a possible history of other autoimmune diseases. In the case of AIP especially type 1, the HISORt criteria has been proposed by the Mayo Clinic to aid in differentiating between AIP and Pancreatic cancer as these two conditions present similarly even though they have very different treatment modalities [23-25]. HISORt stands for:

1. Diagnostic histology: The Mayo clinic has described the following findings as indicative of AIP.

1) A lymphoplasmacytic sclerosing pancreatitis or more than 10 IgG4-positive cells with at least two of the following: periductal lymphoplasmacytic infiltrate, obliterative phlebitis, and acinar fibrosis (type 1 AIP).

2) Idiopathic duct centric pancreatitis or a granulocytic epithelial lesion in the pancreatic duct with minimal IgG4-positive cells in the pancreatic parenchyma (type 2 AIP).

2. Characteristic imaging on computed tomography and/or pancreatography: The main findings that are diagnostic or highly suggestive of AIP are a diffusely enlarged pancreas with featureless borders and delayed enhancement with or without a capsule-like rim [26].

3. Elevated serum IgG4 levels on Serologic testing: A serum concentration of IgG4 that is ≥ 2 times the upper limit of normal is highly suggestive of AIP.

4. Other organ involvement which include the salivary glands (Sjogren's syndrome), bile duct strictures, lung nodules, autoimmune thyroiditis, and kidney (interstitial nephritis with an IgG4-positive plasma cell infiltrate and IgG4 deposits in the tubular basement membrane).

5. Response of pancreatic and extra pancreatic manifestations to glucocorticoid therapy.

Furthermore, EUS, ERCP, or MRCP will reveal multifocal biliary strictures in AIP and diffuse changes in the pancreas in cases of chronic pancreatitis.

## Treatment

Treatment for pancreatic cancer depends upon the stage at diagnosis. Surgery remains the only cure. Only about 15 to 20 percent of patients have resectable disease at the time of diagnosis. Neoadjuvant and adjuvant therapy using chemo/radiotherapy have been tried with surgery with varying results. Invasion or encasement of surrounding vasculature are usually considered absolute contraindications to surgery although there are reports of superior mesenteric vein (SMV) reconstruction in some cases of SMV encasement [27]. Chemotherapy is usually the only treatment modality for distant metastasis. Commonly used agents include 5-Fluorouracil and Gemcitabine.
